# Tungiasis among children in Kenya is associated with poor nutrition status, absenteeism, poor school performance and high impact on quality of life

**DOI:** 10.1371/journal.pntd.0011800

**Published:** 2024-05-22

**Authors:** Lynne Elson, Christopher Kamau, Sammy Koech, Christopher Muthama, George Gachomba, Erastus Sinoti, Elwyn Chondo, Eliud Mburu, Miriam Wakio, Jimmy Lore, Marta Maia, Ifedayo Adetifa, Benedict Orindi, Phillip Bejon, Ulrike Fillinger

**Affiliations:** 1 Kenya Medical Research Institute (KEMRI)-Wellcome Trust, Kilifi, Kenya; 2 Nuffield Department of Medicine, University of Oxford, Oxford, United Kingdom; 3 Department of Health, Muranga, Kenya; 4 Department of Health, Kericho, Kenya; 5 Department of Health, Makueni, Kenya; 6 Department of Health, Nakuru, Kenya; 7 Department of Health, Samburu, Kenya; 8 Department of Health, Kilifi, Kenya; 9 Department of Health, Kajiado, Kenya; 10 Department of Health, Taita Taveta, Kenya; 11 Department of Health, Turkana, Kenya; 12 London School of Hygiene & Tropical Medicine, London, United Kingdom; 13 International Centre for Insect Physiology and Ecology (icipe), Nairobi, Kenya; Hebrew University Hadassah Medical School, ISRAEL

## Abstract

Tungiasis is a highly neglected tropical skin disease caused by the sand flea, *Tunga penetrans*. The flea burrows into the skin inducing a strong inflammatory response, leading to pain and mobility restrictions with potential impacts on quality of life. Few countries implement control efforts and there are few data on the impact of the disease to support policy decisions. We conducted a survey to determine the impact of tungiasis among primary school children across nine counties of Kenya. A total of 10,600 pupils aged 8 to 14 years were randomly selected from 97 primary schools and examined for tungiasis. For 81 cases and 578 randomly selected controls, anthropometric measurements were made, and school attendance and exam scores were collected from school records. Of those with tungiasis, 73 were interviewed regarding their quality of life using a tungiasis-specific instrument. Mixed effect ordered logistic and linear models were used to assess associations between disease status and impact variables. Compared to uninfected pupils, those with tungiasis had lower weight-for-age z-scores (adjusted β -0.41, 95% *CI*: -0.75–0.06, p = 0.020*)*, missed more days of school the previous term (adjusted Incidence Rate Ratio: 1.49, 95% CI: 1.01–2.21, p = 0.046) and were less likely to receive a high score in mathematics (aOR 0.18, 95% CI: 0.08–0.40, p<0.001) and other subjects. Pupils with severe disease (clinical score >10) were four times more likely to experience severe pain than those with mild disease (OR 3.96, 95% CI: 1.35–11.64, p = 0.012) and a higher impact on their quality of life than those with mild disease (aOR 3.57, 95% CI: 1.17–10.8, p = 0.025) when adjusted for covariates. This study has demonstrated tungiasis has a considerable impact on children’s lives and academic achievement. This indicates the need for integrated disease management for school-aged children to protect their physical and cognitive development and their future prospects.

## Author summary

Tungiasis is one of the most neglected tropical diseases (NTDs). It is caused by a flea which burrows into the skin, usually of the feet. The neglect has resulted in large gaps in our knowledge of the disease, how to treat and prevent infection. This is partly due to a lack of appreciation for the impact the disease has on patients’ lives by health officials, policymakers and the research community. In this study we aimed to determine the impact on school children in nine counties of Kenya. We found evidence that children experience considerable pain and itching which affects their ability to attend school and to concentrate on their classes. They had lower exam scores in mathematics, English and science than uninfected children. This study has provided evidence that tungiasis is a disease that has a considerable impact on children and urgently needs more attention from researchers, donors, and policy makers. Integrating tungiasis into comprehensive disease management for school-aged children will protect their physical and cognitive development and their future prospects.

## Introduction

Tungiasis is a highly neglected tropical skin disease which is widespread across sub-Saharan Africa and Latin America [1]. In Kenya, human tungiasis is considered a significant individual and public health threat, with a national prevalence of 1.3% among children aged 8 to 14 years [2]. In selected communities, prevalence can reach as high as 64%, as in north-eastern Uganda [3–6]. Children under 15 years, elderly people and people with disabilities carry the highest disease burden [7].

Tungiasis is caused by female adult sand fleas (*Tunga penetrans*) which penetrate the skin of their mammalian hosts and stay embedded in one spot in the epidermis to mature and produce eggs [8]. The flea causes extensive morbidity resulting from an intense inflammatory response around the rapidly growing sand fleas [9]. The inflammation is further intensified by frequent bacterial superinfection and may result in tetanus, gangrene or septicemia [10]. The vast majority of the embedded sand fleas are located in the feet [1].

Little is known about the impact of tungiasis infection on a patient’s quality of life. The inflammation, pain and itching have been reported to affect children’s ability to sleep [9], walk [9,11], attend school [12] and pay attention in class, thus reducing their school performance [12,13], but this has not been studied systematically. People with tungiasis report being ridiculed and ostracized from their community [14–16]. It was previously demonstrated in a small group of patients in Kenya that tungiasis significantly impacts a child’s Quality of Life using a tungiasis-related Dermatological Quality of Life Index which focuses on a parasitic skin disease of the feet [17].

The objectives of this study were to determine whether tungiasis was associated with children’s nutrition status, school attendance, academic achievement, and quality of life in nine counties of Kenya.

## Methods

### Ethics approval and consent to participate

The study was approved by the Kenya Medical Research Institute (KEMRI) Scientific and Ethics Review Committee (approval number KEMRI/SERU/CGMR-C/170/3895) as well as the Oxford Tropical Research Ethics Committee (reference number 38–19). The study was conducted in accordance with the Helsinki Declaration. During the community engagement phase, a presentation was made to the national Director for Neglected Tropical Diseases at the Ministry of Health, the county and sub-county health management teams and the department of education in all counties to obtain their approval. In each school a meeting was held with the school parent teachers’ association (PTA) or management board to obtain their permission to conduct the survey in their school. The head teacher and PTA chairperson signed the consent form on behalf of the parents and school for the pupils to be examined. Each child gave verbal assent. Community health workers were hired and trained in each school to assist and be the link with the community emphasizing that participation was completely voluntary, and subjects had the opportunity to withdraw from the study at any point in the study.

Informed verbal consent was obtained from parents of pupils selected for interviews using an opt-out methodology. Each pupil was given an information leaflet to take home for their parents along with an opt-out form. Parents were to sign and return the form only if they did not want their child to participate in the interviews, or they could attend the school the next day to clarify any issues they may have. On the following day, if the selected pupils did not have the opt out form and themselves provided verbal assent, they proceeded with the interviews.

All data were collected on PIN protected electronic tablets, stored on password protected RedCap databases on the KEMRI-Wellcome Trust servers. Data were analyzed after export to Excel spreadsheets without inclusion of personal identifiers.

All pupils with tungiasis were referred for treatment to the community health workers or the local health facility using benzyl benzoate provided by the study. For those with secondary bacterial infection and other illnesses requiring treatment, a referral was made to the nearest health facility. When the examination of the 114 pupils was completed, any other pupils in the school who teachers or the pupils themselves thought they might be infected, were allowed to come for an examination, but they were not included in the data collection. They were referred for treatment along with the others.

### Study population

This impact study was a comparison of outcomes among children with and without tungiasis nested in a cross-sectional survey aiming to determine the national level prevalence as reported previously [2]. The survey was conducted in nine counties of Kenya, purposively selected to represent the five major climatic zones, cultures and geography of the country: Turkana, Samburu, Kericho, Nakuru, Muranga, Kajiado, Makueni, Taita Taveta and Kilifi. Within each county 11 primary schools were stratified randomly selected from pre-existing school lists for each sub-county and were surveyed from October 2021 to April 2023.

### Impact study sample size

No other systematic study for these outcomes for tungiasis had been conducted at the time of planning this study so we chose to use a study for another neglected tropical disease to obtain an effect size that we might expect., Consequently, the sample size for the impact assessments was based on a previous study on the impact of *Trichuris* infection on school absenteeism in Jamaica [18]. The Jamaican children who were infected with Trichuris were absent from school for 28% of the school year, while uninfected children were absent for only 12% of the year. If we assume the same would be true for tungiasis in Kenya, the sample size needed to detect this difference between children with tungiasis and uninfected children, with 90% power and 95% confidence interval and a case: control ratio of 1:2, was 102 cases and 208 controls (Open Epi version 3). However, since we did not know if the impact would be as great as was seen for trichuriasis, we applied a design effect of 2 for a total sample of 204 cases and 416 controls. This sample was spread across the targeted 11 schools in each of the nine counties. We aimed to enroll 3 cases and 6 controls in each of 11 schools in each county to a total of 297 cases and 594 controls.

### Selection procedure

Within each selected school an equal number of boys and girls were quasi-randomly selected by asking the pupils to assemble in three age groups; 8 and 9 years; 10 and 11 years; 12 to 14 years, and by gender within each age group. For each of these age/gender groups, every n^th^ pupil (n = total number in the group/ 19) was selected until 19 were selected, giving a total of 114 pupils overall.

The 114 pupils were then asked to assent before their hands and feet were washed and systematically examined for embedded *T*.*penetrans* fleas. Those found infected were assessed for infection intensity (number of live and dead fleas, manipulated lesions and cluster of fleas). Infected pupils were also examined for associated morbidity as described previously [2] recording the presence of symptoms that are easily identified by non-clinical field officers; desquamation, fissures, ulcers and abscess for acute symptoms and hyperkeratosis, deformed nails and lost nails for chronic symptoms. Symptoms normally recorded by previous studies [18] but omitted here since they are difficult for non-clinicians to identify, were oedema, erythema, warmness and peri-ungual hyperkeratosis. Examinations were conducted by non-clinical field workers trained by LE. All infected pupils were also asked how much pain and how much itching they experienced in the past one week from the embedded fleas. They were asked to score this as either none, a little, some or a lot.

In each school, after the 114 pupils had been examined and their tungiasis infection status determined, six uninfected pupils, 3 boys and 3 girls, were stratified randomly selected using the lottery method to enroll the 594 controls required. While it was intended to randomly select 3 infected pupils in each school (for a total of 297), there were too few cases identified so as many infected pupils as possible were enrolled.

### Outcome variables

#### Anthropometrics

Measurements of pupils’ height and weight were taken using a tape measure and simple weighing scales, ensuring they were placed on a flat surface. From these height-for-age and weight-for-age z scores were calculated using the “zanthro” extension for Stata with the UK-WHO Term Growth Charts for adolescents provided [19].

#### School attendance and academic achievement

School attendance and exam records for the previous school term were provided by the teachers and searched for the selected pupils. Data were extracted on the number of days pupils were absent in the whole of the previous term as well as their exam results at the end of the previous school term, for mathematics, English and science. The schools recorded exam scores differently for different school grade. Pupils in grades one to four were given a simple score of 0 to 4, while pupils in grades five to eight were given an actual percentage mark.

**School grade delay** variable was created as follows. First, expected age in years for each school grade; grade 1 being 6.5, grade 2 being 7.5, grade 3 being 8.5 was created. Next, age difference was calculated for each pupil as a pupil’s age minus the expected age for his/her grade. This was then collapsed into a binary variable; 1 for any pupil who was more than 1.0 years older than the expected age for their grade and 0 for all others.

#### Pain and itching

During the foot examinations, only infected pupils were asked to score how much pain and how much itching they experienced in the past one week from the embedded fleas in their feet on a 4-point Likert scale of “none” (= 0), “a little” (= 1), “some” (= 2), and “a lot” (= 3).

#### Quality of life

Only infected pupils were interviewed using the Children’s Dermatological Quality of Life index (CDLQI)[20] previously modified for a skin disease of the feet, the Tungiasis modified Dermatological Quality of Life Index (TLQI) [17] and further modified with two additional questions added. Previous questions were related to mobility, sleep, concentration in school, friendships, bullying and shame (six domains). We added feelings of anger and sadness to a total of eight domains. The instrument asked how much each of these was impacted by the embedded fleas in the past one week, scored on a 4-point Likert scale of “none” (= 0), “a little” (= 1), “some” (= 2), and “a lot” (= 3). The instrument was translated into Kiswahili. The tool was pre-tested and necessary adjustments made prior to the main survey. An overall TLQI score was obtained by summing responses to all questions with a maximum of 24 for each pupil. The scores were then collapsed into quintiles (categories of impact) with thresholds based on a frequency histogram (S3 Table) and Waters et al [21]: 0–1 no impact, 2–5 small impact, 6–10 moderate, 11–15 large, 16–24 very large impact.

### Explanatory variables

The main explanatory variable was disease severity assigned for each infected pupil based on a clinical score as described previously [22]. A total clinical score was calculated for each patient by adding up the number of areas on the feet (each foot being divided in to 9 areas: 5 toes, medial side, lateral side, heel and sole) exhibiting acute symptoms (desquamation, fissures, ulcers and abscess) to a maximum score of 72, and chronic symptoms (hyperkeratosis, deformed nails and lost nails) to a maximum score of 38, to give an overall maximum clinical score of 110. Each pupil was then assigned a severity level: a clinical score less than 11 being a mild case, and a pupil with a clinical score more than 10 being a severe case.

Other explanatory variables were county, school location (urban/ rural), school type (public/ private), pupil age, gender, disability, other skin abnormality, socioeconomic status (SES; as described previously [2]), adults the pupil lives with (both parents/ others), who the main caregiver is (mother/ other), mother’s school level attained (none/ primary/ secondary/ don’t know), father away a lot, mother away a lot, parents attend school meetings (never/sometimes/always), parents make sure pupils completes homework (never/sometimes/always), chronically ill family member, family member disabled, pupil missed school to help at home, pupil sleeps in parents’ house, number of people sleep in room with pupil.

### Data analysis

All analyses were conducted in Stata IC version 15.1 (Stata Corp LLC, College Station, Texas, USA). First, we described the participants by their background characteristics and disease status and tested for differences between the disease groups using Pearson Chi^2^ test or t-tests. Next, univariable mixed effect models were fitted for outcome and all the explanatory variables. Height-for-age and weight-for-age were also included as covariates in models for the other five outcomes. Only covariates with a p-value below 0.200 were included in the multivariable models and stepwise backward elimination used to reach the final models with the lowest Akaike Information Criteria (AIC). Significance of categorical variables in the final model were confirmed using Wald Chi tests. Results for the models are presented in the main body of the manuscript, except for the academic achievement univariable analyses which are presented in the S2 Table. School was included as a random effect in all models. Depending on the nature of the outcome, we fitted either a linear mixed effects model (anthropometrics, academic achievement for upper grade), a negative binomial mixed effects model (absenteeism), a logistic mixed effects model (school grade delay) or ordered logistic mixed effects model (academic achievement for lower grades, pain and itching, and TLQI quintiles). Linear model outcomes are presented as β coefficients with 95% confidence intervals and associated p values. The number of days absent was for a time limit of one term and is therefore an incidence rate. The outcome from the negative binomial model for days absent was exponentiated to obtain incidence rate ratios with 95% confidence intervals and associated p values. The logistic outcomes are presented as odds ratios with 95% confidence intervals and associated p values. In all of the multivariable models the outcomes are then expressed as adjusted ratios.

To identify the quality-of-life domain most affected by tungiasis the percentage of infected pupils scoring a 2 or 3 for each domain was calculated. A two-level ordered logistic regression analysis with school as a random effect was used to test for association of each domain with disease severity.

We performed these analyses under the valid missing at random assumption, as we used likelihood approaches [23].

## Results

### Study population

**Fig 1:**
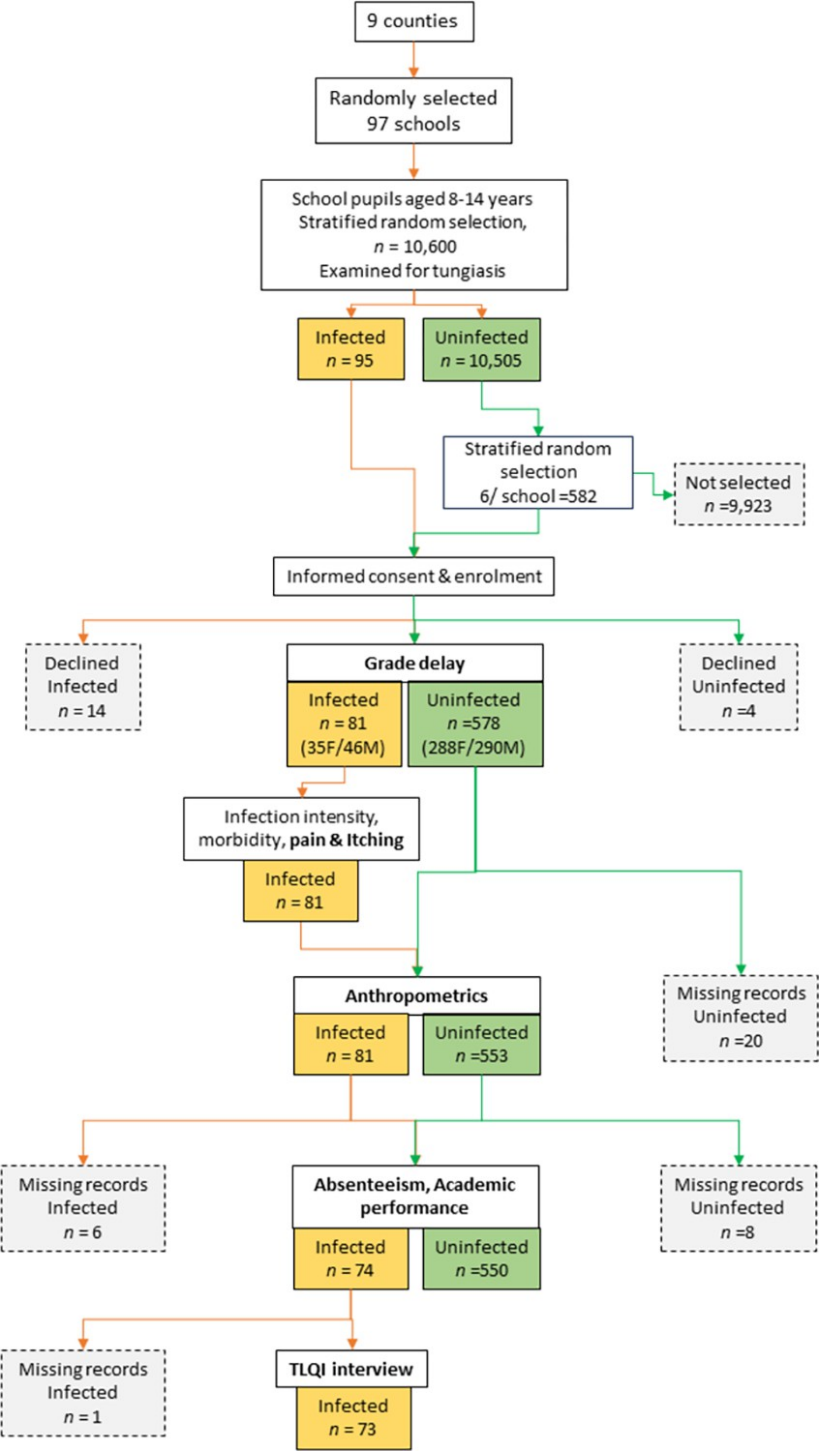
Flow chart to illustrate the number of infected (orange boxes) and uninfected (green boxes) pupils enrolled and with complete data for each outcome (white boxes, bold font). Grey boxes indicate pupils who declined to participate or missing data.

While the study aimed to enroll 99 schools across the nine counties, only 97 were achieved with a total of 659 pupils enrolled. Of these, 578 were uninfected pupils (controls) and 81 were tungiasis infected pupils (cases, Table 1 and Fig 1). The infected pupils were identified in 35 schools across all nine counties. The cases had a median of five embedded fleas (inter-quartile range, IQR 2–15) (Table 1). The majority of these pupils were from public schools (88.8%) and rural areas (87.6%). The mean age was 11 years and 50.1% were boys. Of all the pupils enrolled, 10 (1.5%) were disabled and 42 (6.4%) had some other skin abnormality. There were significantly more infected pupils in public and rural schools than in private and urban schools (Table 1). Additionally, infected pupils came from families with a lower socio-economic status (SES). The flow chart in Fig 1 provides details of the number of participants, infected and uninfected, included or with missing data for each of the study outcomes.

**Table 1:** Characteristics of the study population.

	All pupils	Uninfected	Infected	p
Number of pupils	659	578	81	
Age mean (sd^1^)	11	11.1 (2.0)	10.8 (1.8)	0.234^5^
Gender (% male)	50.1	50.2	56.8	0.265^4^
School type (% public)	88.8	87.2	100	0.001^4^
School location (% rural)	87.6	86.2	97.3	0.004^4^
Disability (%)	1.5	1.2	3.7	0.086^4^
Other skin disease (%)	6.4	5.7	11.1	0.062^4^
SES^2^ (Min 0.45, max 2.41) mean (sd)	1.48 (0.52)	1.53 (0.52)	1.13 (0.42)	<0.001^5^
Number of fleas (median, IQR^3^)	-	-	5 (2–15)	
Clinical score (max 110) (median, IQR)	-	-	4 (1–12)	

^1^ standard deviation

^2^socio-economic status

^3^inter-quartile range

^4^p value for Pearson Chi^2^

^5^ p value from t-test.

### Anthropometrics

Of the 558 uninfected pupils for whom measurements were obtained, 85 (15.2%) were classified as underweight (weight-for-age z-score<-2.0) while 37 (6.6%) were stunted (height-for-age z-score<-2.0). Of the 81 infected pupils, 32 (38.55%) were underweight and 10 (12.05%) were stunted (See S1 Table). Univariable mixed effect linear models for weight-for-age found a significant negative association with tungiasis status (β -0.65, 95% *CI*: -0.99–0.31, p<0.001). This negative association remained (β -0.41, 95% *CI*: -0.75–0.06, p = 0.020)*)* even when adjusted for other possible confounding covariables, including socio-economic status (SES), and whether a child lived with both parents (Table 2). There was no association of tungiasis with height-for-age (β -0.09, 95% CI: -0.47–0.28, p = 0.638).

**Table 2:** Linear mixed effects regression analysis for nutrition status (weight-for-age z-scores).

			UNIVARIABLE				MULTIVARIABLE MODEL			
Variable	Category	N^1^	Effect (β)	95% CI^2^		P^3^	Adjusted effect (β)	95% CI^2^		P^3^
Tungiasis status	Uninfected	553	1				1			
	Infected	81	-0.65	-0.99	-0.31	<0.001	-0.41	-0.75	-0.06	0.020
County	Muranga	89	1							
	Turkana	69	0.13	-0.38	0.65	0.614				
	Samburu	67	-0.56	-1.08	-0.04	0.034				
	Kericho	67	0.16	-0.34	0.67	0.529				
	Nakuru	74	-0.20	-0.70	0.30	0.434				
	Kajiado	73	0.18	-0.32	0.68	0.480				
	Makueni	72	0.03	-0.46	0.52	0.916				
	Taita Taveta	67	0.19	-0.31	0.70	0.454				
	Kilifi	81	-0.86	-1.37	-0.35	0.001				
School type	Public	585	1							
	Private	74	0.57	0.17	0.98	0.005				
School location	Urban	82	1							
	Rural	577	-0.47	-0.88	-0.06	0.024				
Other skin disease	No	617	1							
	Yes	42	-0.21	-0.65	0.24	0.363				
TLQI^4^			0.04	-0.03	0.11	0.245				
Number of days absent last term			0.01	-0.02	0.03	0.627				
SES^5^			0.66	0.43	0.90	<0.001	0.60	0.37	0.83	<0.001
Adults living with	Both Parents	473	1				1			
	Others	186	0.35	0.11	0.58	0.004	0.26	0.03	0.50	0.026
Caregiver	Mother	504	1							
	Others	153	-0.06	-0.30	0.18	0.617				
Mother’s school level	None	110	1							
	Primary	181	0.04	-0.31	0.39	0.827				
	Secondary	254	0.03	-0.32	0.37	0.881				
	Don’t Know	108	-0.32	-0.71	0.07	0.108				
Father away a lot	No	270	1							
	Yes	225	0.00	-0.24	0.23	0.970				
Mother away a lot	No	410	1							
	Yes	173	-0.01	-0.26	0.25	0.960				
Number meals eaten yesterday	1	49	1							
	2	200	-0.01	-0.43	0.42	0.981				
	3	392	0.05	-0.36	0.47	0.802				

^1^ number

^2^confidence interval

^3^ p-value

^4^ tungiasis modified quality of life

^5^ socio-economic status

### Absenteeism

Records for the number of days a pupil was absent during the previous school term were recorded for 624 pupils, of whom 74 were infected. Overall, the number of days missed in the last term were low, median of 1 day (IQR 0–3) for uninfected pupils and 2 days for infected pupils (IQR 0–5). Univariable analyses found pupils with tungiasis missed nearly twice as many days of school than uninfected pupils (IRR: 1.87, 95% *CI*: 1.27–2.75, p = 0.001), but many other covariates were also associated with absenteeism, that could account for this difference (Table 3). When combined in a multivariable model, tungiasis infection was still associated, with infected pupils missing 1.5 times more days than uninfected pupils (aIRR: 1.49, 95% CI: 1.01–2.21, p = 0.046) even when adjusted for county, SES, caregiver school level achieved and family disability (Table 3).

**Table 3:** Negative binomial regression for school absenteeism (number of days absent).

				UNIVARIABLE				MULTIVARIABLE			
Variable	Category	N^1^	Median (IQR^2^)	IRR^3^	95% CI^4^		P^5^	aIRR^6^	95% CI^4^		P^5^
Tungiasis status	No	550	1 (0–2)	1				1			
	Yes	74	2 (0–5.25)	1.87	1.27	2.75	0.001	1.49	1.01	2.21	0.046
County	Taita taveta	61	0 (0–2)	1				1			
	Turkana	69	5 (1–10.5)	4.78	2.35	9.73	<0.001	1.30	0.58	2.92	0.528
	Samburu	64	0 (0–0)	0.31	0.14	0.70	0.005	0.09	0.03	0.22	<0.001
	Kericho	66	1 (0–3.25)	1.41	0.68	2.94	0.352	1.28	0.65	2.50	0.476
	Muranga	86	1 (0–3)	1.56	0.77	3.18	0.22	1.48	0.77	2.83	0.238
	Nakuru	74	1 (0–4)	1.61	0.78	3.31	0.198	1.41	0.73	2.71	0.311
	Kajiado	69	1 (0–3)	1.70	0.82	3.52	0.151	1.59	0.82	3.08	0.169
	Makueni	71	1 (0–3)	1.50	0.73	3.10	0.271	1.17	0.60	2.27	0.643
	Kilifi	64	2 (0–4)	2.54	1.17	5.50	0.018	1.03	0.48	2.20	0.938
School grade/class	8	57	0 (0–1.5)	1							
	1	22	3.5 (1.75–10.5)	6.50	3.00	14.09	<0.001				
	2	80	1 (0–5)	3.79	2.14	6.71	<0.001				
	3	96	1 (0–4)	3.66	2.10	6.40	<0.001				
	4	89	1 (0–3)	2.42	1.36	4.30	0.003				
	5	97	1 (0–4)	2.98	1.70	5.25	<0.001				
	6	90	1 (0–3)	2.53	1.43	4.46	0.001				
	7	93	0 (0–2)	1.96	1.11	3.46	0.021				
Disability	No	615	1 (0–3)	1							
	Yes	9	0 (0–5.5)	1.08	0.40	2.91	0.873				
Other skin disease	No	586	1 (0–3)	1							
	Yes	38	1 (0–3.25)	0.93	0.54	1.58	0.776				
Height-for-age				1.01	0.93	1.11	0.753				
Weight-for-age				1.06	0.95	1.17	0.309				
SES^7^				0.47	0.34	0.65	<0.001	0.50	0.34	0.72	<0.001
Caregiver’s schooling	Some secondary	246	0 (0–2)	1							
	None	100	2 (0–8)	2.27	1.47	3.51	<0.001	2.19	1.28	3.75	0.004
	Primary only	171	1 (0–3)	1.27	0.91	1.75	0.157	1.11	0.80	1.55	0.537
	Don’t know	102	1 (0–4)	1.49	1.01	2.19	0.045	1.38	0.93	2.06	0.112
Family ill some months	No	527	1 (0–3)	1							
	Yes	95	1 (0–3)	0.97	0.68	1.38	0.853				
Family disability	No	584	1 (0–3)	1				1			
	Yes	34	2 (0–6)	1.67	1.00	2.78	0.049	1.69	1.01	2.83	0.044
Source income job	No	480	1 (0–4)								
	Yes	144	0 (0–2)	0.57	0.41	0.80	0.001				
Sleep in parent house	Yes	498	1 (0–3)	1							
	No	125	1 (0–5)	0.84	0.61	1.16	0.288				
Adults living with	Both parents	456	1 (0–3)	1							
	Others	168	1 (0–3.5)	0.91	0.68	1.21	0.505				
Caregiver	Mother	482	1 (0–4)	1							
	Other	141	1 (0–3)	1.00	0.74	1.37	0.978				

^*1*^ number

^*2*^ interquartile range

^*3*^ incidence rate ratio

^*4*^ confidence interval

^*5*^ p-value

^*6*^ adjusted incidence rate ratio

^*7*^ socio-economic status

### Academic performance

Of the 659 pupils, exam results were obtained for 273 from the lower grades (1 to 4) with a median age of 9 years (IQR 8–10.5 years). For these pupils results were scored on a Likert-type scale of 0 to 4. Exam results were also obtained for another 346 pupils from the upper grades (5 to 8) with a median age of 12 years (IQR 11–14 years), for whom actual percentage exam results were obtained.

The percentage of pupils in the lower grades receiving each score for mathematics, English and science are presented in Table 4. Fewer infected pupils received a score of 3 or 4 than uninfected pupils for all subjects.

**Table 4:** Percent of pupils in lower grades achieving each score level for mathematics English and science, by disease status.

Subject	Tungiasis status	N^1^	Exam score					P-value^2^
			0	1	2	3	4	
Mathematics	Uninfected	221	12.2	24.9	39.8	19.9	3.2	<0.001
	Infected	52	34.6	34.6	30.8	0.0	0.0	
English	Uninfected	221	14.9	27.6	34.4	19.0	4.1	<0.001
	Infected	52	34.6	44.2	11.5	9.6	0.0	
Science	Uninfected	221	15.4	24.0	33.9	24.4	2.3	0.001
	Infected	52	32.7	36.5	25.0	5.8	0.0	

^**1**^ number of pupils

^2^ p value for Pearson chi^2^ for infected vs uninfected

To determine whether tungiasis is associated with exam scores in each subject while adjusting for possible confounding variables, panel ordered logistic regression models were run (Table 5). In the lower grades, pupils with tungiasis had five times lower odds of achieving a higher score than uninfected pupils (mathematics aOR 0.18, 95% CI: 0.08–0.40 p<0.001, English aOR 0.20, 95% CI: 0.09–0.46 p<0.001, and science aOR 0.20, 95% CI: 0.09–0.44 p<0.001). The significant confounding variables were disability, the number of days absent from school in the past term, SES, whether the mother was away a lot and the height-for-age z-scores (Table 5). There was no association of school type (public or private) nor weight-for-age z-scores with any subject exam results in this age group.

**Table 5:** Panel Ordered Logistic Regression analysis of school exam results for Mathematics, Science and English for pupils in grade 1 to 4. (Univariable tables in S2 Table).

Variable	Category	N^1^	MATHEMATICS		SCIENCE		ENGLISH	
			aOR^2^ (95% CI^3^)	P^4^	aOR (95% CI)	P	aOR (95% CI)	P
Tungiasis status	Uninfected	219	1		1		1	
	Infected	54	0.18 (0.08–0.39)	<0.001	0.20 (0.09–0.44)	<0.001	0.20 (0.09–0.46)	<0.001
Disability	No	268			1		1	
	Yes	5			0.22 (0.03–1.55)	0.128	0.06 (0.01–0.55)	0.013
Height-for-age			0.84 (0.72–0.99)	0.039	-		-	
Days absent			0.94 (0.89–1.00)	0.049	0.97 (0.91–1.03)	0.252	0.94 (0.87–1.00)	0.067
SES^5^			1.20 (0.63–2.30)	0.649	1.53 (0.77–3.03)	0.222	2.35 (1.17–4.74)	0.017
Mother away a lot	No	167	1		1			
	Yes	74	1.96 (1.03–3.72)	0.040	1.98 (1.02–3.86)	0.045	1.52 (0.78–2.98)	0.220

^*1*^ number

^*2*^ adjusted odds ratio

^*3*^ confidence interval

^*4*^ p-value

^*5*^socio-economic status

The association of school performance with tungiasis was not found for the older pupils in the upper grades, the other independent variables being more strongly associated (S3 Table).

### Delay in school grade

Having to repeat a year or more in the same school grade, and therefore being older than the expected age for that grade (school grade delay) is quite common in Kenya and the possible causes are numerous. Since we found tungiasis is associated with higher absenteeism and academic performance and it has previously been suggested that tungiasis causes grade delay [12], we tested this hypothesis for all enrolled pupils (578 controls and 81 cases). Indeed, overall rates of delay were high, of the 659 pupils enrolled, 38.4% (n = 253) were more than one year older than expected for their school grade, 56.8% (n = 47 of 81) of infected pupils, compared to 35.8% (n = 207 of 578) of uninfected pupils. While school grade delay was associated with tungiasis in the univariable analyses (OR 2.10, 95% *CI*: 1.12–3.94, p = 0.022), several other explanatory covariables were too, and it was these variables that were more strongly associated with school grade delay and tungiasis was not (aOR 1.07, 95% *CI*: 0.52–2.21, p = 0.862) (Table 6).

**Table 6:** Logistic regression analysis for delay in school grade (delayed at least one year).

			UNIVARIABLE				MULTIVARIABLE			
Variable	Category	N^1^	OR^2^	95% CI^3^		P^4^	aOR^5^	95% CI		P
Tungiasis status	Not infected	578	1				1			
	Infected	81	2.10	1.12	3.94	0.022	1.07	0.52	2.21	0.862
County	Muranga	89	1				1			
	Turkana	69	11.44	4.21	31.07	<0.001	6.73	1.82	24.96	0.004
	Samburu	67	3.76	1.40	10.11	0.009	2.76	0.86	8.80	0.087
	Kericho	67	2.09	0.76	5.70	0.152	2.29	0.79	6.67	0.127
	Nakuru	74	1.64	0.60	4.51	0.338	1.33	0.46	3.83	0.602
	Kajiado	73	2.07	0.76	5.60	0.153	2.52	0.86	7.38	0.091
	Makueni	72	1.46	0.54	3.96	0.453	1.34	0.46	3.93	0.592
	Taita Taveta	67	1.29	0.46	3.63	0.636	1.55	0.51	4.68	0.439
	Kilifi	81	20.42	7.12	58.53	<0.001	11.38	3.23	40.08	<0.001
School type	Public	585	1							
	Private	74	0.15	0.05	0.44	0.001				
School location	Urban	82	1							
	Rural	577	3.19	1.20	8.50	0.020				
Disability	No	649	1							
	Yes	10	2.44	0.46	12.90	0.294				
Other skin abnormality	No	617	1							
	Yes	42	1.44	0.64	3.26	0.377				
Height-for-age			0.70	0.60	0.82	<0.001				
Weight-for-age			0.77	0.67	0.89	<0.001	0.67	0.56	0.81	<0.001
Days absent			1.10	1.04	1.17	0.001	1.07	1.01	1.14	0.025
SES^6^			0.22	0.14	0.35	<0.001	0.46	0.25	0.87	0.016
Adults living with	Both parents	473	1							
	Other adults	186	1.14	0.74	1.76	0.543				
Who cares for child	Mother	504	1							
	Others	153	0.75	0.47	1.19	0.219				
Mother’s schooling	None	110	1							
	Primary	181	0.69	0.36	1.33	0.264	2.04	0.88	4.74	0.095
	Secondary	254	0.56	0.28	1.10	0.092	2.20	0.90	5.34	0.082
	Don’t know	108	0.69	0.33	1.47	0.341	1.74	0.67	4.51	0.255
Father away a lot	No	270	1							
	Yes	228	0.96	0.61	1.52	0.875				
Mother away a lot	No	410	1							
	Yes	173	0.99	0.60	1.64	0.972				
Parents attend school meetings	Never	38	1							
	Sometimes	260	1.50	0.66	3.42	0.338				
	Always	358	0.89	0.38	2.06	0.781				
Parents check homework done	Never	95	1							
	Sometimes	235	0.94	0.52	1.70	0.834				
	Always	328	0.44	0.24	0.81	0.008				
Family member ill some months	No	548	1							
	Yes	108	1.06	0.63	1.78	0.824				
Family member has disability	No	616	1							
	Yes	37	1.52	0.66	3.49	0.326				
Miss school to help at home	No	548	1							
	Yes	102	2.14	1.25	3.65	0.005	1.51	0.81	2.79	0.191
Sleep in parents’ house	No	133	1							
	Yes	524	0.64	0.39	1.04	0.072				

^*1*^ number

^*2*^ odds ratio

^*3*^ confidence interval

^*4*^ p-value

^*5*^ adjusted odds ratio

^*6*^ socio-economic status

### Experience of pain, itching and quality of life

Only infected pupils were interviewed for this part of the study and comparisons were between mild and severe cases, as summarized in Table 7. While 81 pupils responded to the pain and itching questions (58 mild, 23 severe), only 73 cases for the quality-of-life interview (51 mild, 22 severe). The only significant difference between the mild and severe cases was their infection intensity, by study design.

**Table 7:** Characteristics of disease severity groups.

	Mild disease	Severe disease	P value
Number of pupils	58	23	
Age mean (sd^1^)	11 (2.0)	10.4 (1.3)	0.182^5^
Gender (% male)	55.2	60.9	0.641^4^
Disability (%)	3.5	4.3	0.847^4^
Other skin disease (%)	6.9	21.7	0.055^4^
SES^2^ (Min 0.45, max 2.41) mean (sd)	1.15 (0.44)	1.06 (0.37)	0.398^5^
Number of fleas (median, IQR^3^)	3 (2–6)	22 (12–52)	<0.001^5^
Clinical score (max 110) (median, IQR)	2 (0–4)	22 (14–38)	

^1^ standard deviation, ^2^socio-economic status, ^3^inter-quartile range ^4^p value for Pearson Chi^2^, ^5^ p value from t-test.

### Pain and itching

A total of 81 infected pupils were asked about the amount of pain they experienced from the embedded fleas and the amount of itching the fleas caused them in the previous one week, scored from 0 (= none) to 3 (= a lot). Univariable models demonstrated that pupils with severe tungiasis were nearly four times more likely to experience higher levels of pain than the pupils with mild tungiasis (OR 3.96, 95% CI 1.35–11.64, p = 0.012). The same was true for itching (OR 3.81, 95% CI 1.30–11.14, p = 0.014).

### Quality of life

Full records for the quality-of-life interview were available for 73 infected pupils, 51 with mild and 22 with severe disease. The domains of the Tungiasis-modified Dermatological Life Quality Index (TLQI) with the most pupils expressing “some” (3) or “a lot” (4) of impact were shame, disturbed sleep and difficulty concentrating at school due to the itching (Table 8). Using panel ordered univariable models it was clear mild cases felt the same levels of shame as severe cases. However, pupils with severe tungiasis had four times higher odds of experiencing higher levels of impact on concentration and mobility (Table 8). Although the p-values were not less than 0.05, pupils with severe disease had 3 times higher odds of experiencing higher levels of bullying and disturbed sleep.

**Table 8:** Association of Tungiasis associated-quality-of-life domains for pupils with severe disease compared to pupils with mild disease.

Quality of Life domain	% scoring “some” or “a lot” of impact	Odds Ratio	95% Conf. Interval		P-value
Shame	41.8	1.15	0.31	4.20	0.836
Disturbed sleep	35.8	2.70	0.96	7.54	0.059
Difficulty concentrating due to itch	32.8	4.19	1.06	16.48	0.041
Feel anger	31.3	1.25	0.42	3.75	0.690
Mobility	25.4	4.15	1.29	13.36	0.017
Bullying	23.9	3.43	0.94	12.54	0.062
Affects friendship	28.4	0.79	0.27	2.34	0.674
Feel sad	28.4	1.37	0.42	4.47	0.602

**Fig 2:**
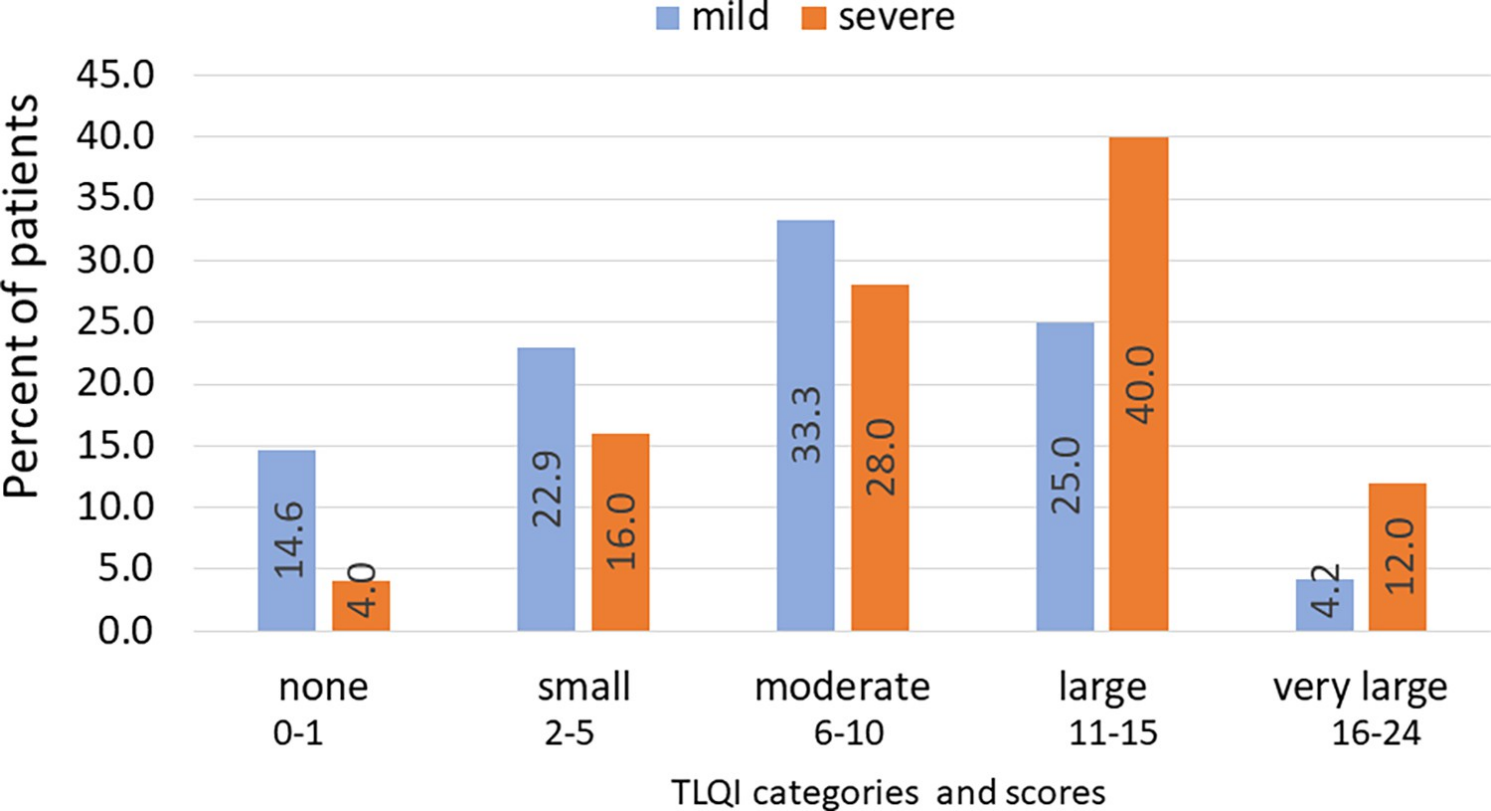
Distribution of tungiasis-associated quality-of-life categories by disease severity. Severe disease: orange bars, mild disease: blue bars.

When all of the domain scores were summed for each pupil to obtain the TLQI score, the median score was 9 (IQR 4–13) and ranged from 0 to 24 (the maximum possible). When collapsed into quintiles, 80% of severe cases reported a moderate to very large impact on quality of life (TLQI 6–24), while 62.5% of mild cases were considered to have this level of impact (Fig 2). Since quality of life could be impacted by many factors in a child’s life, we conducted multivariable analysis with the same set of explanatory variables as used above with the TLQI quintiles. We found pupils with severe tungiasis had a nearly four times higher odds of experiencing a higher category of impact than those with mild tungiasis (aOR 3.57, 95% *CI*: 1.17–10.8) even when adjusted for the only variable which remained significant in the model, the mother being away a lot (Table 9). Children whose mothers were away a lot had three times higher odds of experiencing a higher category of impact.

**Table 9:** Panel Ordered Logistic Regression analysis for tungiasis-associated Quality of Life (TLQI) quintiles (categories) for tungiasis patients.

			Univariable				Multivariable			
Variable	Category	N^1^	OR^2^	95% CI^3^		P^4^	aOR^5^	95% CI		P
Disease severity	Mild	51	1				1			
	Severe	22	3.61	0.97	13.39	0.055	3.57	1.17	10.8	0.025
County	Muranga	19	1							
	Turkana	4	0.50	0.04	6.12	0.585				
	Samburu	6	3.08	0.25	37.68	0.378				
	Kericho	4	4.09	0.20	83.67	0.360				
	Nakuru	8	3.84	0.38	39.14	0.256				
	Kajiado	4	4.28	0.31	59.15	0.277				
	Makueni	5	1.24	0.07	20.59	0.881				
	Taita Taveta	1	-	-	-	-				
	Kilifi	22	0.44	0.06	3.06	0.403				
Height-for-age			1.09	0.82	1.46	0.543				
Weight-for-age			1.18	0.82	1.68	0.375				
SES^6^			1.26	0.28	5.61	0.764				
Adults living with	Both parents	54	1							
	Other adults	19	0.93	0.30	2.85	0.894				
Who cares for child	Mother	59	1							
	Others	14	1.28	0.34	4.79	0.709				
Family member ill some months	No	548	1							
	Yes	108	0.55	0.13	2.34	0.416				
Family disability	No	616	1							
	Yes	37	0.48	0.03	8.81	0.618				
Father away a lot	No	33	1							
	Yes	27	1.69	0.53	5.36	0.376				
Mother away a lot	No	51	1				1			
	Yes	21	3.45	1.14	10.45	0.029	3.41	1.18	9.85	0.023
Mother’s schooling	Some secondary	21	1							
	None	7	0.34	0.03	3.43	0.359				
	Primary only	26	1.45	0.36	5.91	0.604				
	Don’t know	18	2.61	0.50	13.77	0.258				
Source income from job	No	67	1							
	Yes	10	0.55	0.11	2.86	0.481				
Miss school to help	No	62	1							
	Yes	13	0.41	0.11	1.48	0.172				
Sleep in parent house	No	11	1							
	Yes	66	2.82	0.75	10.56	0.125				
# People sleep in same room			1.11	0.99	1.25	0.072				

^*1*^ number

^*2*^ odds ratio

^*3*^ confidence interval

^*4*^ p-value

^*5*^ adjusted odds ratio

^*6*^ socio-economic status

## Discussion

Few of the countries endemic for tungiasis have a national control strategy. This is in part due to lack of data on the disease burden and evidence-based interventions, but also a lack of evidence for the impact the disease has on those who are infected. Here we attempted to quantify the impact tungiasis has on nutrition status, school attendance, academic achievement, grade delay and quality of life.

The study found that tungiasis infection is negatively associated with weight-for-age, with higher levels of school absenteeism and lower exam scores among pupils in the younger grades. Children with severe tungiasis had higher levels of pain and itching, and a reduced quality of life, particularly as regards mobility, concentration in school and disturbed sleep, than children with mild tungiasis.

The most striking impact identified in this study was the very poor academic achievement of infected pupils in grade one to four in all three exam subjects compared to uninfected peers, even when adjusted for other factors that can influence school performance, including absenteeism which was independently associated with tungiasis. These findings confirm those from one previous descriptive study in Kenya which alluded to higher levels of absenteeism, repeating a school year and school dropout among children with tungiasis [12] and a study in Rwanda which suggested pupils with tungiasis had higher absenteeism and lower exam scores [13]. Our findings on lower academic achievement aligns with what has been reported for other diseases. Previously children with repeated episodes of malaria [24,25] or with helminth infections [26,27] have been found to have lower school attendance, learning ability, memory and academic achievement.

Another factor that could be a confounder in the association with school performance is malnutrition, low height-for-age (stunting) and weight-for-age (underweight), both of which have previously been associated with poor academic achievement [28–30]. However, in the current study only height-for-age was positively associated with mathematics scores in the lower grades in this study and did not confound the association with tungiasis, so we find it likely that tungiasis independently affects children’s school performance.

Stunting is thought to be caused by chronic under-nutrition and is associated with poor neurological and cognitive development and therefore learning ability and academic achievement [31,32]. It is possible that chronic pain leads to poor nutritional intake, but it is also possible that poverty is a causal factor that confounds the association between nutrition and tungiasis. The mechanism of action for tungiasis infection on poor school performance needs to be explored further but could be related to the inflammation induced by the embedded fleas causing extreme pain and itching as demonstrated in this study, particularly children with severe disease. This in turn affects mobility, sleep, concentration in class, and social exclusion as seen in the quality-of-life assessments and ability to attend school, which could all impact cognitive development and learning. In fact findings of ours from another study demonstrated school children with tungiasis did have impaired cognitive development compared to their uninfected peers [33].

**Fig 3:**
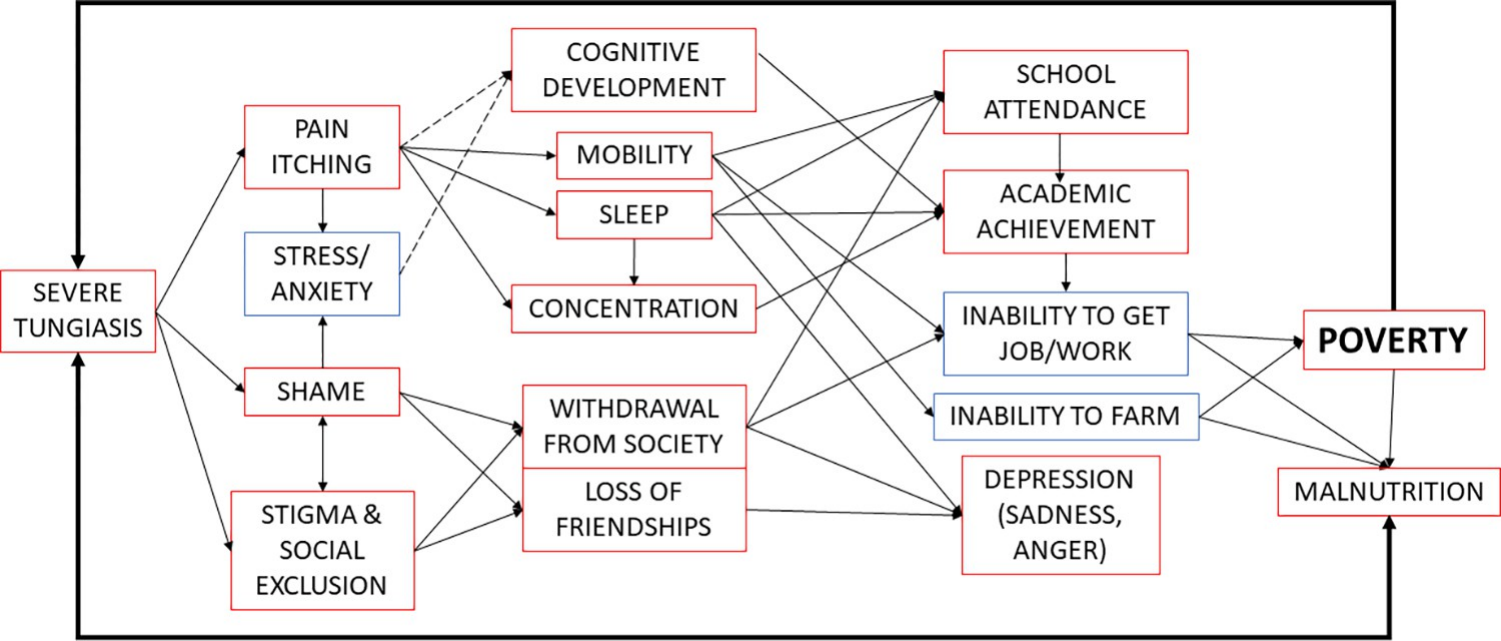
A conceptual framework of the impacts of tungiasis trapping people in poverty. Red boxes documented impacts, blue boxes anecdotal reports, solid arrows: likely pathway, broken arrow: possible pathway.

All of these factors are interconnected in a vicious cycle as illustrated in Fig 3, possibly trapping children into a life of poverty [34] which puts them at greater risk of tungiasis. The association of tungiasis with low weight-for-age even when adjusted for SES, i.e. not confounded by poverty, needs further investigation but may be a result of this cascade. The embedded fleas and the associated itching and mental state may affect a child’s appetite, or the fleas could be depriving the host of essential nutrients, particularly those with a high infection intensity.

The proportion of infected pupils (68.5%) who reported a moderate to very high impact on their TLQI was similar to the previous small tungiasis study in Kenya (78%) [17] and a study of cutaneous larva migrans in Brazil (71%) [35], which were higher than that reported for scabies (45%) [36], suggesting tungiasis has a higher impact on the quality of life of children than does scabies, another skin disease caused by an arthropod. Each of these studies used a disease-specific, modified version of the general CDLQI [21]. Both the previous and current Kenyan tungiasis studies identified sleep disturbance and concentration in school to be commonly affected domains which have been shown to lead to poor cognitive development [37], learning and memory [38] as well as depression [39]. Both studies also identified feelings of shame to be common, reflecting the impact of stigma and discrimination which is common in endemic communities, where people with tungiasis are often labelled as dirty, lazy and careless [14–16]. In fact, 23% and 28% of infected pupils in this study experienced high levels of bullying and affected friendships. Interventions such as those piloted in western Kenya recently by Mørkve and Munkejord [40] deserve further exploration to reduce stigma and improve psychological well-being and health-seeking behavior [41].

Recently a new classification of disease severity for tungiasis was proposed based on the correlation of clinical symptoms with infection intensity [22] and we found it to fit well with the cases found in this survey of nine counties in Kenya [2]. The distribution of patients on the correlation suggested a threshold of a clinical score of 10, roughly equivalent to 10 fleas to create two disease groups, mild and severe. In the past, other researchers have used a three-tier classification (mild 1–5 fleas, moderate 6–30 fleas and severe >30 fleas [5]), but this was not based on a systematic correlation with clinical signs. The fact that children with the new classification of severe tungiasis in this randomly selected population from multiple geographic areas and cultures across Kenya expressed four times higher levels of pain and itching and a higher impact on quality of life, mobility and concentration, confirms this is a clinically relevant classification for future use in other studies, and for targeting and monitoring interventions.

There were several possible limitations to this study. Firstly, the main limitation was the low number of cases identified during the survey resulting in the need to adapt the study design to enroll every infected pupil identified rather than randomly selecting three from all infected pupils in every school. It is noteworthy that despite this low number of cases, significant associations were still able to be identified with several outcomes.

One limitation of the academic outcome is the possibility that the scoring system for this age group by teachers could be subjective and biased. However, the schools were selected randomly and were from nine different counties across Kenya and it is unlikely all the teachers would have the same bias. Using the school as a random effect in the models should also control for this. If this association reflects a negative bias of teachers against pupils with tungiasis rather than an actual difference in academic achievement, that would also be an important outcome and barrier to these children’s development which would require interventions. Further studies to investigate this might be warranted.

Another limitation is that participants were selected from children who were attending school on the day of the surveys. The most heavily infected children in the community may have been absent from school on that day on account of the disease and may miss more days of school due to their infection. If we had enrolled such children, we would expect to have seen even higher impacts on absenteeism rates and even lower exam scores.

The lack of association of tungiasis with exam scores in the older age group (grade 5 to 8) may well be the result of the very small number of cases enrolled in the study. Ideally this study should be repeated in public schools only and enrolling an equal number of cases and controls, across all school grades into a longitudinal study to determine whether older pupils also have lower exam scores and whether the impact seen at the younger age affects achievement in later years.

Lastly, although we examined the pupils for other skin diseases, we did not test them for diseases such as malaria and intestinal helminths which could have confounded several of our outcomes including absenteeism, school grade delay and school performance. However, the considerably larger number of controls and their random selection from randomly selected schools across the nine counties in the study should have gone some way to counter this.

In conclusion, our study has demonstrated that tungiasis has considerable impact on many aspects of the lives and development of children, just as has been documented for malaria and helminth infections. Diagnosis, surveillance, disease management and stigma reduction for children with tungiasis urgently need to be integrated into the existing platforms created for these other diseases, in line with the WHO Road map for Neglected Tropical Diseases [42]. Only by addressing all the diseases impacting these marginalized children will they have an equal opportunity to develop alongside their uninfected peers.

## Supporting information

S1 Table Table of participant distribution by covariates and disease status. (DOCX)

S2 Table Univariable Regression tables for pupil exam results in Mathematics, English and Science. (DOCX)

S3 Table Multivariable Regression tables for pupil exam results in Mathematics, English and Science for pupils in the upper grades (5 to 8). (DOCX)

S1 Fig Frequency histogram of TLQI with quintile thresholds. (DOCX)
