# Supplementary material for: Tungiasis among children in Kenya is associated with poor nutrition status, absenteeism, poor school performance and high impact on quality of life
Source: PLoS Negl Trop Dis. 2024 May 22;18(5):e0011800. doi: 10.1371/journal.pntd.0011800 (PMC11149845; doi:10.1371/journal.pntd.0011800)
Supplement: S1 Table — (DOCX) [file pntd.0011800.s001.docx]

## **S1 Table. Participant Distribution by covariates and disease status**

| **DEPENDENT/ OUTCOME VARIABLES** | | | | | |
| --- | --- | --- | --- | --- | --- |
| **Variable** | **type** | **Missing**  **n** | **n** | **Uninfected**  **n** | **Infected**  **n** |
| Total population |  |  | 659 | 578 | 81 |
| Height-for-age z-score (mean/sd) | Numeric | 21 | 638 | 558  (-0.10, 1.5) | 80  (-0.12, 1.85) |
| Stunted | Binary |  | 47 | 37 | 10 |
| Weight-for-age z-score (mean/sd) | Numeric | 26 | 633 | 553  (-0.77, 1.30) | 80  (-1.63, 1.44) |
| Underweight | Binary |  | 117 | 85 | 32 |
| Number of days absent last term (median/ IQR) | Numeric | 35 | 624 | 551  (1,0-3) | 73  (2, 0-5) |
| Mathematics grade 1-4 (median, IQR) | Categorical, Ordinal | 30 | 273 | 221  (2, 1-2) | 52  (1, 0-2) |
| English grade 1-4 (median, IQR) | Categorical, Ordinal | 30 | 273 | 221  (2, 1-2) | 52  (1, 0-1) |
| Science grade 1-4 (median, IQR) | Categorical, Ordinal | 30 | 273 | 221  (2, 1-3) | 52  (1, 0-2) |
| Mathematics grade 5-8  (mean, sd) | Numeric | 5 | 346 | 326  (46.3/ 18.4) | 20  (36.9/ 15.7) |
| English grade 5-8 (mean/sd) | Numeric | 5 | 346 | 326  (48.9/ 17.5) | 20  (40.2/ 18.1) |
| Science grade 5-8 (mean/sd) | Numeric | 5 | 345 | 325  (51.5/ 18.2) | 20  (38.8/ 21.5) |
| Grade delay  (> 1 year, %) | Binary | 0 | 659 | 578  (207, 35.8%) | 81  (46, 56.8%) |
| Pain (mild/severe) (median, IQR) | Categorical, Ordinal | 0 | 80 | - | 57 (0, 0-1) /  22 (1, 0-3) |
| Itching (mild/severe) (median, IQR) | Categorical, Ordinal | 0 | 80 | - | 57 (0, 0-1)/  22 (2, 0-3) |
| TmDLQI (mild/severe) (median (IQR) | Categorical, Ordinal | 10 | 73 | - | 51 (8, 4-11)/  22 (12, 8-14) |

| **INDEPENDENT COVARIATES** | | | | | |
| --- | --- | --- | --- | --- | --- |
| **Variable** | **categories** | **Missing**  **n** | **Total**  **n** | **Uninfected**  **n** | **Infected**  **n (%)** |
| County | Muranga | 0 | 89 | 68 | 21 (23.6) |
|  | Turkana |  | 69 | 65 | 4 (5.8) |
|  | Samburu |  | 67 | 61 | 6 (9.0) |
|  | Kericho |  | 67 | 63 | 4 (6.0) |
|  | Nakuru |  | 74 | 66 | 8 (10.8) |
|  | Kajiado |  | 73 | 69 | 4 (5.5) |
|  | Makueni |  | 72 | 67 | 5 (6.9) |
|  | Taita Taveta |  | 67 | 66 | 1 (1.5) |
|  | Kilifi |  | 81 | 53 | 28 (34.6) |
| school type | public | 0 | 585 | 504 | 81 (14.2) |
|  | private |  | 74 | 74 | 0 (0) |
| school location | urban | 0 | 82 | 80 | 2 (2.4) |
|  | rural |  | 577 | 498 | 79 (13.7) |
| Age |  | 2 | 657 | 576 | 81 (12.3) |
| sex | female | 0 | 323 | 288 | 35 (10.8) |
|  | male |  | 336 | 290 | 46 (13.7) |
| disability | no | 0 | 649 | 571 | 78 (12.0) |
|  | yes |  | 10 | 7 | 3 (30.0) |
| other skin abnormality | no | 0 | 617 | 545 | 72 (11.7) |
|  | yes |  | 42 | 33 | 9 (21.4) |
| SES Quintiles | 1 | 49 | 109 | 84 | 25 (22.9) |
|  | 2 |  | 124 | 101 | 23 (18.6) |
|  | 3 |  | 124 | 114 | 10 (8.1) |
|  | 4 |  | 132 | 122 | 10 (7.6) |
|  | 5 |  | 121 | 119 | 2 (1.7) |
| Adults living with | Both parents | 0 | 473 | 414 | 59 (12.5) |
|  | Other adults |  | 186 | 164 | 22 (11.8) |
| Who cares for child | Mother | 2 | 504 | 440 | 64 (12.7) |
|  | Others |  | 153 | 136 | 17 (11.1) |
| Mother's schooling | none | 6 | 110 | 102 | 8 (7.3) |
|  | primary |  | 181 | 153 | 28 (15.5) |
|  | secondary |  | 254 | 230 | 24 (9.5) |
|  | don't know |  | 108 | 88 | 20 (18.5) |
| Father away a lot | No | 161 | 270 | 235 | 35 (12.9) |
|  | Yes |  | 228 | 201 | 27 (11.8) |
| Mother away a lot | No | 76 | 410 | 357 | 53 (12.9) |
|  | yes |  | 173 | 151 | 22 (12.7) |
| parents attend school meetings | never | 3 | 38 | 31 | 7 (18.4) |
|  | sometimes |  | 260 | 221 | 39 (15.0) |
|  | always |  | 358 | 323 | 35 (9.8) |
| parents check homework done | never | 1 | 95 | 79 | 16 (16.8) |
|  | sometimes |  | 235 | 195 | 40 (17.0) |
|  | always |  | 328 | 303 | 25 (7.6) |
| Family member ill some months | no | 3 | 548 | 482 | 66 (12.0) |
|  | yes |  | 108 | 93 | 15 (13.9) |
| Family member has disability | No | 0 | 616 | 541 | 75 (12.2) |
|  | Yes |  | 37 | 33 | 4 (10.8) |
| miss school to help at home | No | 9 | 548 | 483 | 65 (11.9) |
|  | Yes |  | 102 | 88 | 14 (13.7) |
| sleep in parents' house | No | 2 | 133 | 120 | 13 (9.8) |
|  | Yes |  | 524 | 456 | 68 (13.0) |
| Family income from a job | No | 0 | 509 | 438 | 71 (14.0) |
|  | yes |  | 150 | 140 | 10 (6.7) |
| Number of people sleep in same room (mean, sd) |  | 5 | 654 | 573 | 81 |
| Number of meals eaten yesterday | 1 | 18 | 49 | 40 | 9 (18.4) |
|  | 2 |  | 200 | 171 | 29 (14.5) |
|  | 3 |  | 392 | 355 | 37 (9.4) |
|  |  |  |  |  |  |
|  |  |  |  |  |  |
