# Supplementary material for: Tungiasis among children in Kenya is associated with poor nutrition status, absenteeism, poor school performance and high impact on quality of life
Source: PLoS Negl Trop Dis. 2024 May 22;18(5):e0011800. doi: 10.1371/journal.pntd.0011800 (PMC11149845; doi:10.1371/journal.pntd.0011800)
Supplement: S2 Table — (DOCX) [file pntd.0011800.s002.docx]

**S2 Table. Univariable Regression tables for pupil exam results in Mathematics, Science and English**

Contents

[Univariable regression analyses for exam scores for pupils in grade 1 to 4. 1](#_Toc143594792)

[Maths score 1](#_Toc143594793)

[Science score 3](#_Toc143594794)

[English score 4](#_Toc143594795)

[Univariable regression analyses for exams results for pupils in grade 5 to 8. 6](#_Toc143594796)

[**Maths** result (%) 6](#_Toc143594797)

[**English** result (%) 7](#_Toc143594798)

[**Science** result (%) 9](#_Toc143594799)

## Univariable regression analyses for exam scores for pupils in grade 1 to 4.

| Mathematics score |  | N | OR | 95% CI | | P |
| --- | --- | --- | --- | --- | --- | --- |
| Tunga status | uninfected | 219 | 1 |  |  |  |
|  | infected | 54 | 0.21 | 0.11 | 0.42 | <0.001 |
| county | Muranga | 65 | 1 |  |  |  |
|  | Turkana | 26 | 0.73 | 0.26 | 2.06 | 0.548 |
|  | Samburu | 17 | 0.46 | 0.14 | 1.53 | 0.205 |
|  | Kericho | 23 | 2.71 | 0.85 | 8.66 | 0.094 |
|  | Nakuru | 49 | 4.46 | 1.76 | 11.28 | 0.002 |
|  | Kajiado | 35 | 2.88 | 1.06 | 7.81 | 0.038 |
|  | Makueni | 17 | 0.82 | 0.26 | 2.59 | 0.733 |
|  | Taita Taveta | 8 | 0.92 | 0.17 | 4.93 | 0.922 |
|  | Kilifi | 33 | 0.69 | 0.24 | 1.95 | 0.483 |
| school type | private | 28 | 1 |  |  |  |
|  | public | 245 | 0.24 | 0.09 | 0.68 | 0.007 |
| school location | urban | 20 | 1 |  |  |  |
|  | rural | 243 | 0.22 | 0.08 | 0.57 | 0.002 |
| Age |  |  | 1.04 | 0.86 | 1.26 | 0.671 |
| sex | girls | 115 | 1 |  |  |  |
|  | boys | 158 | 0.95 | 0.59 | 1.53 | 0.823 |
| disability | no | 268 | 1 |  |  |  |
|  | yes | 5 | 0.14 | 0.02 | 1.13 | 0.065 |
| other skin disease | no | 247 | 1 |  |  |  |
|  | yes | 26 | 1.43 | 0.62 | 3.34 | 0.402 |
| Weight-for-age z-score |  |  | 0.98 | 0.82 | 1.17 | 0.804 |
| Height-for-age z-score |  |  | 0.88 | 0.76 | 1.02 | 0.084 |
| days absent |  |  | 0.92 | 0.87 | 0.96 | 0.001 |
| SES quintiles | 5 | 43 | 1 |  |  |  |
|  | 1 | 50 | 0.39 | 0.15 | 1.02 | 0.056 |
|  | 2 | 53 | 0.22 | 0.09 | 0.55 | 0.001 |
|  | 3 | 51 | 0.73 | 0.3 | 1.78 | 0.49 |
|  | 4 | 55 | 0.85 | 0.38 | 1.92 | 0.696 |
| adults living with | both parents | 202 | 1 |  |  |  |
|  | others | 71 | 0.93 | 0.53 | 1.63 | 0.794 |
| who cares for pupil | mother | 208 | 1 |  |  |  |
|  | others | 65 | 0.94 | 0.52 | 1.70 | 0.844 |
| number siblings |  |  | 0.96 | 0.85 | 1.07 | 0.441 |
| mother school level | None | 37 | 1 |  |  |  |
|  | primary | 69 | 2.1 | 0.88 | 4.99 | 0.093 |
|  | secondary | 105 | 2.35 | 1 | 5.54 | 0.051 |
|  | Don’t know | 60 | 1.16 | 0.46 | 2.91 | 0.756 |
| father away a lot | no | 106 | 1 |  |  |  |
|  | yes | 100 | 1.24 | 0.7 | 2.2 | 0.471 |
| mother away a lot | no | 167 | 1 |  |  |  |
|  | yes | 74 | 2.1 | 1.11 | 3.95 | 0.022 |
| parents attend school meetings | never | 22 | 1 |  |  |  |
|  | sometimes | 109 | 1.54 | 0.63 | 3.81 | 0.346 |
|  | always | 141 | 1.54 | 0.62 | 3.86 | 0.353 |
| parent help with homework | never | 51 | 1 |  |  |  |
|  | sometimes | 103 | 1.17 | 0.58 | 2.35 | 0.668 |
|  | always | 119 | 2.24 | 1.12 | 4.5 | 0.023 |
| family member ill for months | no | 241 | 1 |  |  |  |
|  | yes | 32 | 0.68 | 0.33 | 1.39 | 0.289 |
| family member has disability | no | 261 | 1 |  |  |  |
|  | yes | 10 | 0.6 | 0.18 | 1.98 | 0.402 |
| family income from job | no | 227 | 1 |  |  |  |
|  | yes | 46 | 2.18 | 1.14 | 4.18 | 0.019 |
| sleep in parent house | no | 44 | 1 |  |  |  |
|  | yes | 228 | 0.86 | 0.43 | 1.7 | 0.665 |
| number of meals yesterday | 1 | 20 | 1 |  |  |  |
|  | 2 | 83 | 1.68 | 0.59 | 4.84 | 0.334 |
|  | 3 | 170 | 3.92 | 1.37 | 11.19 | 0.011 |
| Miss school to help parents | No | 227 |  |  |  |  |
|  | yes | 43 | 0.47 | 0.24 | 0.92 | 0.028 |

| Science score |  | Odds Ratio | 95% CI | | P |
| --- | --- | --- | --- | --- | --- |
| Tunga status | uninfected | 1 |  |  |  |
|  | infected | 0.25 | 0.13 | 0.49 | 0 |
| county | Muranga | 1 |  |  |  |
|  | Turkana | 0.58 | 0.18 | 1.91 | 0.374 |
|  | Samburu | 0.37 | 0.10 | 1.39 | 0.14 |
|  | Kericho | 3.07 | 0.89 | 10.55 | 0.075 |
|  | Nakuru | 4.19 | 1.48 | 11.86 | 0.007 |
|  | Kajiado | 5.15 | 1.65 | 16.12 | 0.005 |
|  | Makueni | 1.95 | 0.56 | 6.83 | 0.298 |
|  | Taita Taveta | 1.79 | 0.29 | 10.96 | 0.528 |
|  | Kilifi | 1.79 | 0.57 | 5.64 | 0.321 |
| school type | private | 1 |  |  |  |
|  | public | 3.15 | 0.99 | 10.09 | 0.053 |
| school location | urban | 1.00 |  |  |  |
|  | rural | 0.36 | 0.12 | 1.09 | 0.072 |
| Age |  | 1.03 | 0.86 | 1.22 | 0.757 |
| age_difference |  | 0.93 | 0.72 | 1.21 | 0.579 |
| sex | girls | 1 |  |  |  |
|  | boys | 1.00 | 0.62 | 1.62 | 0.987 |
| disability | no | 1.00 |  |  |  |
|  | yes | 0.22 | 0.03 | 1.66 | 0.141 |
| other skin disease | no | 1.00 |  |  |  |
|  | yes | 0.76 | 0.32 | 1.82 | 0.535 |
| Weight-for-age z-score |  | 1.04 | 0.87 | 1.24 | 0.662 |
| Height-for-age z-score |  | 0.95 | 0.81 | 1.11 | 0.491 |
| days absent |  | 0.95 | 0.90 | 1.00 | 0.038 |
| SES quintiles | 5 | 1 |  |  |  |
|  | 1 | 1.99 | 0.88 | 4.51 | 0.1 |
|  | 2 | 2.27 | 0.90 | 5.75 | 0.083 |
|  | 3 | 3.96 | 1.59 | 9.82 | 0.003 |
|  | 4 | 4.59 | 1.66 | 12.69 | 0.003 |
| adults living with | both parents | 1 |  |  |  |
|  | others | 0.68 | 0.38 | 1.21 | 0.188 |
| who cares | mother | 1 |  |  |  |
|  | others | 0.83 | 0.46 | 1.52 | 0.555 |
| number siblings |  | 1.04 | 0.92 | 1.17 | 0.498 |
| mother school level | none | 1 |  |  |  |
|  | primary | 2.27 | 0.90 | 5.70 | 0.082 |
|  | secondary | 2.36 | 0.93 | 5.99 | 0.071 |
|  | don’t know | 1.43 | 0.53 | 3.84 | 0.475 |
| father away a lot | no | 1 |  |  |  |
|  | yes | 0.68 | 0.37 | 1.27 | 0.229 |
| mother away a lot | no | 1 |  |  |  |
|  | yes | 1.92 | 1.00 | 3.66 | 0.049 |
| parents attend school meetings | never | 1 |  |  |  |
|  | sometimes | 2.39 | 0.91 | 6.28 | 0.077 |
|  | always | 3.25 | 1.20 | 8.76 | 0.02 |
| parent help with homework | never | 1 |  |  |  |
|  | sometimes | 1.41 | 0.67 | 2.96 | 0.363 |
|  | always | 1.57 | 0.74 | 3.33 | 0.238 |
| family member ill for months | no | 1 |  |  |  |
|  | yes | 0.74 | 0.35 | 1.57 | 0.432 |
| family member has disability | no | 1 |  |  |  |
|  | yes | 0.98 | 0.29 | 3.28 | 0.971 |
| sleep in parent house | no | 1 |  |  |  |
|  | yes | 0.88 | 0.44 | 1.76 | 0.717 |
| miss school to help parents | no | 1 |  |  |  |
|  | yes | 0.43 | 0.21 | 0.87 | 0.019 |
| sleep on | bed | 1 |  |  |  |
|  | floor | 0.56 | 0.29 | 1.06 | 0.074 |
| number of meals yesterday | 1 | 1 |  |  |  |
|  | 2 | 1.37 | 0.44 | 4.29 | 0.585 |
|  | 3 | 2.72 | 0.87 | 8.53 | 0.086 |

| English score |  | OR | 95% CI | | P |
| --- | --- | --- | --- | --- | --- |
|  |  |  |  |  |  |
| Tunga status | uninfected |  |  |  |  |
|  | infected | 0.25 | 0.13 | 0.48 | <0.001 |
| county | Muranga |  |  |  |  |
|  | Turkana | 0.92 | 0.34 | 2.46 | 0.867 |
|  | Samburu | 0.60 | 0.19 | 1.88 | 0.382 |
|  | Kericho | 4.20 | 1.44 | 12.20 | 0.008 |
|  | Nakuru | 3.05 | 1.27 | 7.35 | 0.013 |
|  | Kajiado | 2.96 | 1.14 | 7.67 | 0.025 |
|  | Makueni | 1.06 | 0.35 | 3.24 | 0.915 |
|  | Taita Taveta | 1.25 | 0.28 | 5.58 | 0.773 |
|  | Kilifi | 0.61 | 0.23 | 1.59 | 0.313 |
| school type | private | 5.03 | 1.93 | 13.13 | 0.001 |
|  | public |  |  |  |  |
| school location | urban |  |  |  |  |
|  | rural | 3.70 | 1.49 | 9.22 | 0.005 |
| Age |  | 1.16 | 0.97 | 1.37 | 0.098 |
| sex | girls | 0.88 | 0.55 | 1.41 | 0.604 |
|  | boys |  |  |  |  |
| disability | no |  |  |  |  |
|  | yes | 0.08 | 0.01 | 0.66 | 0.019 |
| other skin disease | no |  |  |  |  |
|  | yes | 0.96 | 0.41 | 2.23 | 0.923 |
| Weight-for-age z-score |  | 1.02 | 0.85 | 1.21 | 0.858 |
| Height-for-age z-score |  | 0.95 | 0.82 | 1.09 | 0.491 |
| days absent |  | 0.91 | 0.87 | 0.96 | 0.001 |
| SES quintiles | 5 |  |  |  |  |
|  | 1 | 0.16 | 0.06 | 0.41 | 0 |
|  | 2 | 0.22 | 0.09 | 0.53 | 0.001 |
|  | 3 | 0.39 | 0.17 | 0.90 | 0.028 |
|  | 4 | 0.61 | 0.27 | 1.35 | 0.223 |
| adults living with | both parents |  |  |  |  |
|  | others | 0.66 | 0.38 | 1.15 | 0.145 |
| who cares | mother |  |  |  |  |
|  | others | 0.63 | 0.35 | 1.14 | 0.129 |
| number siblings |  | 1.01 | 0.91 | 1.13 | 0.856 |
| mother school level | 0 |  |  |  |  |
|  | 1 | 1.58 | 0.69 | 3.65 | 0.281 |
|  | 2 | 2.39 | 1.05 | 5.45 | 0.039 |
|  | 9 | 0.91 | 0.37 | 2.20 | 0.826 |
| father away a lot | no |  |  |  |  |
|  | yes | 1.02 | 0.57 | 1.81 | 0.956 |
| mother away a lot | no |  |  |  |  |
|  | yes | 1.45 | 0.77 | 2.73 | 0.246 |
| parents attend school meetings | never |  |  |  |  |
|  | sometimes | 0.93 | 0.38 | 2.26 | 0.869 |
|  | always | 1.89 | 0.77 | 4.59 | 0.162 |
| parent help with homework | never |  |  |  |  |
|  | sometimes | 0.96 | 0.47 | 1.97 | 0.912 |
|  | always | 1.76 | 0.86 | 3.60 | 0.124 |
| family member ill for months | no |  |  |  |  |
|  | yes | 0.62 | 0.30 | 1.27 | 0.189 |
| family member has disability | no |  |  |  |  |
|  | yes | 0.53 | 0.16 | 1.72 | 0.289 |
| family income from job | no |  |  |  |  |
|  | yes | 3.34 | 1.78 | 6.29 | 0 |
| sleep in parent house | no |  |  |  |  |
|  | yes | 0.78 | 0.40 | 1.52 | 0.458 |
| number of meals yesterday | 1 |  |  |  |  |
|  | 2 | 0.41 | 0.14 | 1.18 | 0.099 |
|  | 3 | 1.53 | 0.55 | 4.28 | 0.415 |

## Univariable regression analyses for exams results for pupils in grade 5 to 8.

| **Mathematics** result (%) |  | N | Coef. | 95% CI | | P |
| --- | --- | --- | --- | --- | --- | --- |
| Tungiasis status | uninfected | 326 |  |  | |  |
|  | infected | 20 | -10.06 | -18.47 | -1.65 | 0.019 |
| county | Muranga | 23 |  |  |  |  |
|  | Turkana | 43 | 0.80 | -11.06 | 12.66 | 0.895 |
|  | Samburu | 48 | 1.91 | -9.77 | 13.59 | 0.749 |
|  | Kericho | 42 | 9.22 | -2.60 | 21.04 | 0.126 |
|  | Nakuru | 25 | 3.88 | -8.69 | 16.46 | 0.545 |
|  | Kajiado | 31 | 4.24 | -8.15 | 16.63 | 0.503 |
|  | Makueni | 54 | 1.89 | -9.35 | 13.12 | 0.742 |
|  | Taita Taveta | 49 | 4.71 | -7.03 | 16.44 | 0.432 |
|  | Kilifi | 31 | -1.82 | -14.87 | 11.23 | 0.784 |
| School type | Public | 301 |  |  |  |  |
|  | Private | 45 | 21.43 | 14.56 | 28.30 | <0.001 |
| School location | Urban | 45 |  |  |  |  |
|  | rural | 301 | -9.37 | -17.20 | -1.55 | 0.019 |
| Age |  |  | -0.77 | -1.96 | 0.42 | 0.203 |
| sex | girls | 191 |  |  |  |  |
|  | boys | 155 | 0.59 | -2.78 | 3.96 | 0.731 |
| disability | no | 342 |  |  |  |  |
|  | yes | 4 | -3.83 | -19.42 | 11.77 | 0.631 |
| other skin disease | no | 335 |  |  |  |  |
|  | yes | 11 | -5.14 | -14.95 | 4.67 | 0.304 |
| Height-for-age |  |  | 0.36 | -1.04 | 1.76 | 0.616 |
| Weight-for-age |  |  | 0.47 | -1.08 | 2.01 | 0.555 |
| Days absent |  |  | -0.71 | -1.31 | -0.12 | 0.019 |
| SES |  |  | 10.81 | 6.70 | 14.93 | <0.001 |
| adults living with | both parents | 249 |  |  |  |  |
|  | others | 97 | 2.19 | -1.66 | 6.04 | 0.264 |
| who cares for pupil | mother | 274 |  |  |  |  |
|  | others | 71 | 1.86 | -2.36 | 6.08 | 0.388 |
| Mother school | None | 65 |  |  |  |  |
|  | primary | 98 | -0.87 | -6.59 | 4.85 | 0.766 |
|  | secondary | 138 | 6.09 | 0.37 | 11.81 | 0.037 |
|  | Don’t know | 42 | 2.91 | -3.92 | 9.73 | 0.404 |
| Father away a lot | no | 145 |  |  |  |  |
|  | Yes | 118 | 1.75 | -2.36 | 5.86 | 0.404 |
| Mother away a lot | no | 226 |  |  |  |  |
|  | Yes | 88 | 0.07 | -4.24 | 4.37 | 0.975 |
| Parent attend school meetings | never | 14 |  |  |  |  |
|  | sometimes | 137 | 2.29 | -7.17 | 11.74 | 0.635 |
|  | always | 194 | 6.85 | -2.70 | 16.40 | 0.16 |
| Parent assist homework | never | 40 |  |  |  |  |
|  | sometimes | 122 | 1.32 | -4.88 | 7.51 | 0.677 |
|  | always | 183 | 6.00 | -0.22 | 12.22 | 0.058 |
| Family ill months | no | 282 |  |  |  |  |
|  | yes | 62 | -3.53 | -8.11 | 1.04 | 0.13 |
| Family disability | no | 318 |  |  |  |  |
|  | Yes | 24 | -3.48 | -10.28 | 3.32 | 0.316 |
| source income job | no | 249 |  |  |  |  |
|  | Yes | 97 | 6.56 | 2.43 | 10.68 | 0.002 |
| Miss school help at home | no | 288 |  |  |  |  |
|  | Yes | 53 | -5.98 | -10.86 | -1.09 | 0.017 |
| Sleep parent house | no | 81 |  |  |  |  |
|  | Yes | 265 | 1.89 | -2.34 | 6.12 | 0.38 |

| **English** result (%) |  | N | Coef. | 95% CI | | P |
| --- | --- | --- | --- | --- | --- | --- |
| Tungiasis status | uninfected | 326 |  |  |  |  |
|  | infected | 20 | -6.25 | -14.77 | 2.26 | 0.15 |
| county | Muranga | 23 |  |  |  |  |
|  | Turkana | 43 | -11.74 | -21.16 | -2.33 | 0.014 |
|  | Samburu | 48 | -8.16 | -17.39 | 1.07 | 0.083 |
|  | Kericho | 42 | 3.12 | -6.29 | 12.52 | 0.516 |
|  | Nakuru | 25 | -2.28 | -12.53 | 7.98 | 0.664 |
|  | Kajiado | 31 | 1.54 | -8.40 | 11.48 | 0.761 |
|  | Makueni | 54 | 4.41 | -4.62 | 13.44 | 0.339 |
|  | Taita Taveta | 49 | 0.93 | -8.33 | 10.18 | 0.845 |
|  | Kilifi | 31 | -9.42 | -19.60 | 0.76 | 0.07 |
| School type | Public | 301 |  |  |  |  |
|  | Private | 45 | 18.57 | 12.51 | 24.63 | <0.001 |
| School location | Urban | 45 |  |  |  |  |
|  | rural | 301 | -10.54 | -17.32 | -3.75 | 0.002 |
| Age |  |  | -2.48 | -3.67 | -1.30 | <0.001 |
| sex | girls | 191 |  |  |  |  |
|  | boys | 155 | -2.64 | -6.11 | 0.83 | 0.136 |
| disability | no | 342 |  |  |  |  |
|  | yes | 4 | -7.78 | -24.00 | 8.43 | 0.347 |
| other skin disease | no | 335 |  |  |  |  |
|  | yes | 11 | 0.44 | -9.66 | 10.54 | 0.932 |
| Height-for-age |  |  | 0.78 | -0.63 | 2.20 | 0.276 |
| Weight-for-age |  |  | 1.55 | 0.01 | 3.09 | 0.048 |
| Days absent |  |  | -0.43 | -1.05 | 0.19 | 0.176 |
| SES |  |  | 13.93 | 10.32 | 17.54 | <0.001 |
| adults living with | both parents | 249 |  |  |  |  |
|  | others | 97 | 3.49 | -0.45 | 7.42 | 0.083 |
| who cares for pupil | mother | 274 |  |  |  |  |
|  | others | 71 |  |  |  |  |
| Mother school | None | 65 |  |  |  |  |
|  | primary | 98 | 3.31 | -2.30 | 8.91 | 0.247 |
|  | secondary | 138 | 10.62 | 5.01 | 16.23 | <0.001 |
|  | Don’t know | 42 | 7.51 | 0.76 | 14.25 | 0.029 |
| Father away a lot | no | 145 |  |  |  |  |
|  | Yes | 118 | -0.42 | -4.74 | 3.89 | 0.847 |
| Mother away a lot | no | 226 |  |  |  |  |
|  | Yes | 88 | -1.12 | -5.45 | 3.21 | 0.611 |
| Parent attend school meetings | never | 14 |  |  |  |  |
|  | sometimes | 137 | 4.72 | -4.85 | 14.28 | 0.334 |
|  | always | 194 | 9.18 | -0.41 | 18.77 | 0.061 |
| Parent assist homework | never | 40 |  |  |  |  |
|  | sometimes | 122 | 3.17 | -2.92 | 9.27 | 0.308 |
|  | always | 183 | 11.59 | 5.53 | 17.66 | <0.001 |
| Family ill months | no | 282 |  |  |  |  |
|  | yes | 62 | -3.09 | -7.78 | 1.60 | 0.197 |
| Family disability | no | 318 |  |  |  |  |
|  | Yes | 24 | -4.20 | -11.17 | 2.77 | 0.238 |
| source income job | no | 249 |  |  |  |  |
|  | Yes | 97 | 6.98 | 2.84 | 11.13 | 0.001 |
| Miss school help at home | no | 288 |  |  |  |  |
|  | Yes | 53 | -5.78 | -10.89 | -0.66 | 0.027 |
| Sleep parent house | no | 81 |  |  |  |  |
|  | Yes | 265 | 1.45 | -2.86 | 5.76 | 0.511 |

| **Science** result (%) |  | N | Coef. | 95% CI | | P |
| --- | --- | --- | --- | --- | --- | --- |
| Tungiasis status | uninfected | 326 |  |  | |  |
|  | infected | 20 | -8.83 | -17.54 | -0.11 | 0.047 |
| county | Muranga | 23 |  |  |  |  |
|  | Turkana | 43 | -3.70 | -15.27 | 7.86 | 0.53 |
|  | Samburu | 48 | -1.76 | -13.14 | 9.61 | 0.762 |
|  | Kericho | 42 | 6.17 | -5.36 | 17.70 | 0.294 |
|  | Nakuru | 25 | -1.98 | -14.30 | 10.34 | 0.753 |
|  | Kajiado | 31 | -0.88 | -13.01 | 11.24 | 0.886 |
|  | Makueni | 54 | 4.20 | -6.80 | 15.21 | 0.454 |
|  | Taita Taveta | 49 | 4.33 | -7.10 | 15.75 | 0.458 |
|  | Kilifi | 31 | -4.29 | -16.97 | 8.39 | 0.507 |
| School type | Public | 301 |  |  |  |  |
|  | Private | 45 | 20.63 | 13.80 | 27.45 | <0.001 |
| School location | Urban | 45 |  |  |  |  |
|  | rural | 301 | -8.08 | -15.86 | -0.31 | 0.041 |
| Age |  |  | -0.25 | -1.49 | 0.99 | 0.692 |
| sex | girls | 191 |  |  |  |  |
|  | boys | 155 | 1.56 | -1.94 | 5.06 | 0.382 |
| disability | no | 342 |  |  |  |  |
|  | yes | 4 | -6.18 | -22.41 | 10.04 | 0.455 |
| other skin disease | no | 335 |  |  |  |  |
|  | yes | 11 | 0.80 | -9.40 | 10.99 | 0.878 |
| Height-for-age |  |  | 0.16 | -1.25 | 1.57 | 0.826 |
| Weight-for-age |  |  | 0.50 | -1.08 | 2.09 | 0.535 |
| Days absent |  |  | -0.55 | -1.17 | 0.07 | 0.081 |
| SES |  |  | 11.08 | 6.95 | 15.21 | <0.001 |
| adults living with | both parents | 249 |  |  |  |  |
|  | others | 97 | 1.82 | -2.17 | 5.82 | 0.371 |
| who cares for pupil | mother | 274 |  |  |  |  |
|  | others | 71 | 0.21 | -4.20 | 4.62 | 0.925 |
| Mother school | None | 65 |  |  |  |  |
|  | primary | 98 | -4.05 | -9.87 | 1.77 | 0.173 |
|  | secondary | 138 | 5.85 | 0.00 | 11.70 | 0.05 |
|  | Don’t know | 42 | 1.03 | -5.94 | 8.00 | 0.773 |
| Father away a lot | no | 145 |  |  |  |  |
|  | Yes | 118 | 4.13 | -0.03 | 8.29 | 0.052 |
| Mother away a lot | no | 226 |  |  |  |  |
|  | Yes | 88 | 0.42 | -3.92 | 4.76 | 0.849 |
| Parent attend school meetings | never | 14 |  |  |  |  |
|  | sometimes | 137 | 6.61 | -3.11 | 16.34 | 0.183 |
|  | always | 194 | 10.75 | 0.94 | 20.57 | 0.032 |
| Parent assist homework | never | 40 |  |  |  |  |
|  | sometimes | 122 | 2.32 | -4.01 | 8.65 | 0.473 |
|  | always | 183 | 9.45 | 3.11 | 15.78 | 0.003 |
| Family ill months | no | 282 |  |  |  |  |
|  | yes | 62 | -0.32 | -5.12 | 4.49 | 0.897 |
| Family disability | no | 318 |  |  |  |  |
|  | Yes | 24 | -1.96 | -8.97 | 5.06 | 0.585 |
| source income job | no | 249 |  |  |  |  |
|  | Yes | 97 | 10.12 | 5.97 | 14.27 | <0.001 |
| Miss school help at home | no | 288 |  |  |  |  |
|  | Yes | 53 | -4.21 | -9.29 | 0.86 | 0.104 |
| Sleep parent house | no | 81 |  |  |  |  |
|  | Yes | 265 | 4.78 | 0.45 | 9.12 | 0.031 |
