# Supplementary material for: Tungiasis among children in Kenya is associated with poor nutrition status, absenteeism, poor school performance and high impact on quality of life
Source: PLoS Negl Trop Dis. 2024 May 22;18(5):e0011800. doi: 10.1371/journal.pntd.0011800 (PMC11149845; doi:10.1371/journal.pntd.0011800)
Supplement: S3 Table — (DOCX) [file pntd.0011800.s003.docx]

S3 Table. Multivariable linear regression analysis of school exam results in mathematics, science and English for pupils in grades 5 to 8. (see Univariable tables in S2).

|  |  | N^1^ | MATHEMATICS | | SCIENCE | | ENGLISH | |
| --- | --- | --- | --- | --- | --- | --- | --- | --- |
| Variable | Category |  | β (95% CI^2^) | P^3^ | β (95% CI) | P | β (95% CI) | P |
| Tungiasis status | Uninfected | 326 | 1 |  | 1 |  | 1 |  |
|  | Infected | 20 | -8.52 (-17.03− -0.02) | 0.050 | -6.03 (-15.45−3.40) | 0.210 | -4.57 (-12.20−3.07) | 0.241 |
| School type | Public | 301 | 1 |  | 1 |  | 1 |  |
|  | Private | 45 | 15.39 (7.73−23.04) | <0.001 | 18.06 (8.82−27.30) | <0.001 | 8.96 (3.17−14.74) | 0.002 |
| School location | Urban | 45 |  |  | 1 |  |  |  |
|  | Rural | 301 |  |  | 8.81 (-0.22−17.84) | 0.056 |  |  |
| Age |  |  |  |  |  |  | -2.72 (-3.91− -1.54) | <0.001 |
| Weight-for-age |  |  |  |  |  |  | -1.18 (-2.74−0.38) | 0.138 |
| Days absent |  |  |  |  | -0.49 (-1.14−0.16) | 0.142 | -0.51 (-1.10−0.08) | 0.091 |
| SES^4^ |  |  | 4.07 (-1.03−9.16) | 0.117 | 3.80 (-2.10−9.70) | 0.206 | 7.47 (2.44−12.50) | 0.004 |
| Adults living with | Both parents | 249 |  |  |  |  | 1 |  |
|  | Other | 97 |  |  |  |  | 4.04 (0.25−7.84) | 0.037 |
| Mother schooling level | None | 65 | 1 |  | 1 |  | 1 |  |
|  | Primary | 98 | -5.38 (-11.37−0.61) | 0.078 | -5.74 (-12.44−0.96) | 0.093 | -0.69 (-6.37−4.98) | 0.811 |
|  | Secondary | 138 | -1.46 (-7.90−4.99) | 0.658 | 0.18 (-7.23−7.59) | 0.962 | 1.39 (-4.71−7.49) | 0.655 |
|  | Don’t know | 42 | -1.20 (-8.08−5.67) | 0.732 | 2.05 (-5.97−10.07) | 0.616 | 0.71 (-5.80−7.22) | 0.831 |
| Father away a lot | No | 145 |  |  | 1 |  |  |  |
|  | Yes | 118 |  |  | 2.08 (-1.87−6.03) | 0.302 |  |  |
| Parents attend school meetings | Never | 14 | 1 |  |  |  |  |  |
|  | Sometimes | 137 | 2.11 (-7.57−11.79) | 0.669 |  |  |  |  |
|  | Always | 194 | 4.41 (-5.50−14.31) | 0.383 |  |  |  |  |
| Parent make sure do homework | Never | 40 | 1 |  | 1 |  | 1 |  |
|  | Sometimes | 122 | 1.23 (-5.08−7.54) | 0.702 | 3.43 (-3.87−10.74) | 0.357 | 3.85 (-2.18−9.88) | 0.211 |
|  | Always | 183 | 2.19 (-4.52−8.91) | 0.522 | 8.92 (1.62−16.23 | 0.017 | 6.90 (0.76−13.03) | 0.028 |
| Family member ill some months | No | 282 | 1 |  |  |  | 1 |  |
|  | Yes | 62 | -3.58 (-8.08−0.93) | 0.119 |  |  | -2.05 (-6.60−2.51) | 0.378 |
| Miss school to help parents | No | 288 | 1 |  | 1 |  | 1 |  |
|  | Yes | 53 | -4.02 (-9.17−1.13) | 0.126 | -1.25 (-7.32−4.81) | 0.685 | -3.11 (-8.41−2.20) | 0.251 |
| Sleep in parent house | No | 81 |  |  | 1 |  |  |  |
|  | Yes | 265 |  |  | 4.99 (0.31−9.68) | 0.037 |  |  |

^1^ number, ^2^ confidence interval, ^3^ p-value, ^4^ socio-economic status
