# Supplementary material for: Tungiasis among children in Kenya is associated with poor nutrition status, absenteeism, poor school performance and high impact on quality of life
Source: PLoS Negl Trop Dis. 2024 May 22;18(5):e0011800. doi: 10.1371/journal.pntd.0011800 (PMC11149845; doi:10.1371/journal.pntd.0011800)

## **S1 Fig. Frequency histogram of TLQI for all patients.**

Red lines indicate thresholds for quintile grouping.


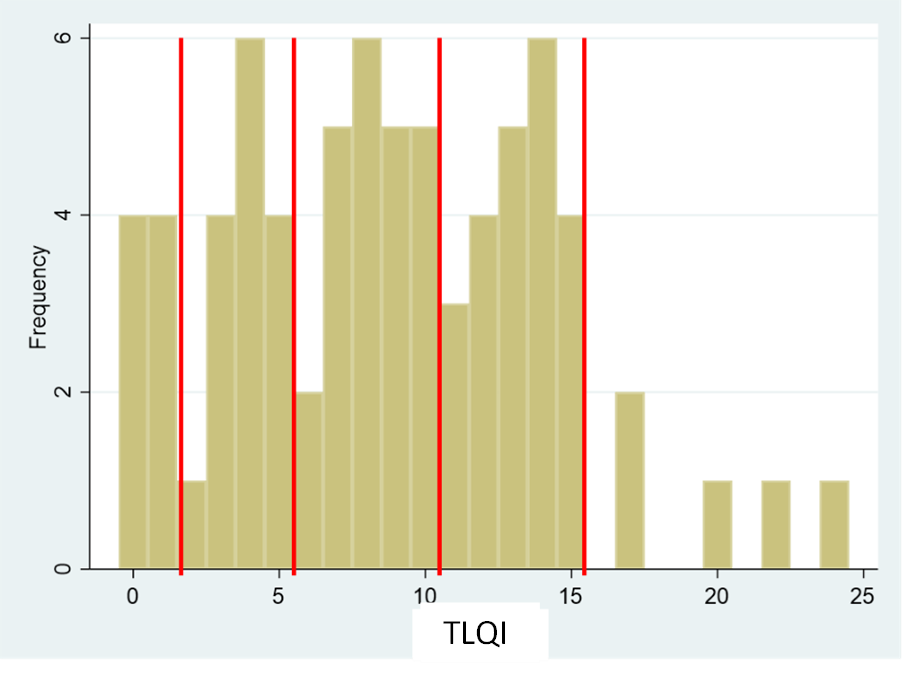

Supplement: S1 Fig — (DOCX) [file pntd.0011800.s004.docx]
